# 

**Published:** 1916-08

**Authors:** 


### Publishers' Announcement

Thoughts of valuable importance appear in the pages of THE DENTAL REGISTER each month, and it is essential that a professional man read the compositions of our liberal and learned contributors.

Dentistry has great educational features, and the dentist who recognizes this and devotes himself to the scientific research side of his work as well as to the business end, develops a combination in himself that produces success and reputation. To the educated mind there is no dull moment, no time in which there is nothing to do; each minute is worth something and can be well spent. Time is the asset given free to every one. How it is spent determines the calibre of each one to use it for all it is worth.

THE DENTAL REGISTER has maintained its conservative and proper form of journalism throughout seventy years and has a following it is proud of. It voices the workers—the men and women who are earnest and sincere—those who desire to make use of the precious moments given them. To all whose heart rings with earnest aspirations THE DENTAL REGISTER will appeal.

### ANNOUNCEMENTS

ST. LOUIS DENTAL SOCIETY. St. Louis enjoys the distinction of having one of the oldest active dental societies in the world, and the oldest dental college west of Ohio. In this environment many of the eminent men in our profession have developed. With pardonable pride in this record the members of the St. Louis Dental Society are preparing to celebrate the sixtieth anniversary of its organization, November 16, 17 and 18, 1916.

A series of scientific papers along advanced line of present interest will be presented by the foremost teachers and original investigators. Individual clinicians and group clinics by societies and study clubs are solicited to accurately delineate the modern progress of dentistry.

A selected exhibit of dental appliances, equipment and reputable pharmaceutical preparations will be displayed, but anticipate obtaining something more valuable than a collection of samples.

You shall not be permitted to forget the date—November 16th, 17th and 18th. Plan to represent your society on this program.

---

THE CHAYES DENTAL CLUB. On June 15, 1916, the Chayes Dental Club held its first meeting at the Hotel McAlpin, New York. It is composed of forty dentists who have adopted the Chayes system of movable, removable bridgework in their practices, and have banded together for study and mutual help and to further propagate the idea among their fellow practitioners.

It is proposed at subsequent meetings to hold open discussion on the all-important subject of bridgework, and to conduct clinics demonstrating the beneficial effect of Chayes bridgework in the human mouth.

The society invites correspondence with dentists in New York and other cities who are doing the work or are interested in it.

President, Dr. Philip Hussa, 8 West Fortieth St., New York; Secretary, Dr. J. O. Lief, 320 Fifth Ave., New York.

